# Retraction: screening and identification of a renal carcinoma specific peptide from a phage display peptide library

**DOI:** 10.1186/1756-9966-31-21

**Published:** 2012-03-13

**Authors:** Xiangan Tu, Jintao Zhuang, Wenwei Wang, Liang Zhao, Liangyun Zhao, Jiquan Zhao, Chunhua Deng, Shaopeng Qiu, Yuanyuan Zhang

**Affiliations:** 1 Department of Urology, The First Affiliated Hospital, Sun Yat-sen University, Guangzhou 510700, Guangdong, PR China; 2 Wake Forest Institute for Regenerative Medicine, Wake Forest University Health Sciences, Winston-Salem, NC, 27157, USA

## Retraction

The authors would like to retract the article "Screening and Identification of a Renal Carcinoma Specific Peptide from a Phage Display Peptide Library" [1]. After the article was published it was revealed that the data in figure three, reported to show the Immunohistochemical staining of renal carcinoma, actually shows the results from an unrelated experiment on lung tissue due to incorrect labeling. The authors apologize to the readers, reviewers, and editors for publishing this erroneous data.
